# Assessing a modified-AJCC TNM staging system in the New South Wales Cancer Registry, Australia

**DOI:** 10.1186/s12885-019-6062-x

**Published:** 2019-08-28

**Authors:** Sheena Lawrance, Chau Bui, Vidur Mahindra, Maria Arcorace, Claire Cooke-Yarborough

**Affiliations:** Cancer Institute NSW, PO Box 41, Alexandria, Sydney, NSW 1435 Australia

**Keywords:** Cancer Registries, Cancer, Oncology, Epidemiology, Registry, Stage, TNM Staging

## Abstract

**Background:**

In 2017, the New South Wales Cancer Registry (NSWCR) participated in a project, supported by Cancer Australia, aiming to provide national stage data for melanoma, prostate, colorectal, breast, and lung cancers diagnosed in 2011. Simplified business rules based on the American Joint Committee for Cancer (AJCC) Tumour-Node-Metastasis (TNM) stage were applied to obtain Registry-Derived (RD) stage, defined as the best estimate of TNM stage at diagnosis using routine notifications available within cancer registries. RD-stage was compared with Degree of Spread (DoS), which has been recorded for all applicable cancers in NSWCR at a population-based level since 1972, and a summary AJCC-TNM stage group, which has been collected variably since 2006. For each of the five high incidence cancers, we compared the level of improvements RD-staging provided in terms of completeness and accuracy (alignment to more clinically relevant AJCC-TNM) over DoS.

**Methods:**

For each of the five cancers, stage data were extracted from NSWCR pre- and post- RD-staging to compare data completeness across all three staging systems. The alignment between DoS/RD-stage and AJCC-TNM was compared, as were the expected and observed cross-tabulated frequency distributions using a subset of NSWCR data. To determine differences between use of DoS, RD-stage, and AJCC-TNM in an epidemiological analysis, we compared survival models developed from each of the three stage variables.

**Results:**

We found RD-staging provided greatest stage data completeness and alignment to AJCC-TNM for prostate cancers, followed by breast, then melanoma and lung cancers. For colorectal cancer, summary stage from DoS was confirmed as an equivalent surrogate staging system to both AJCC-TNM and RD-stage.

**Conclusions:**

This analysis provides an evidence-based approach that can be used to inform decision-making for resource planning and potential implementation of a new stage data field in population-based cancer registries.

**Electronic supplementary material:**

The online version of this article (10.1186/s12885-019-6062-x) contains supplementary material, which is available to authorized users.

## Background

Cancer staging is an important clinical tool to determine prognosis and treatment plans. In population-based cancer studies, stage at initial diagnosis is important for understanding cancer outcomes and guiding cancer control activities [1, 2]. Stage is recorded variably in different Population-based Cancer Registries (PBCRs), which hinders comparisons or consolidation of stage data for population analyses across different PBCR jurisdictions [2, 3].

The American Joint Committee for Cancer (AJCC) Tumour Node Metastasis (TNM) stage classification system, hereafter referred to as AJCC-TNM, is the staging system most commonly used in clinical practice [4]. The AJCC-TNM Stage Group (AJCC-SG) represents a synthesis of values based on the size and extent of the primary tumour (T), degree of spread to local lymph nodes (N), and level of metastasis (M) according to tumour-specific algorithms, which may also factor in non-anatomic values. AJCC-TNM data are either not reported or under-reported in most PBCRs in low and middle income countries, and in some well-established PBCRs from high-income jurisdictions (such as Australia), AJCC-TNM data have been found to be defined and reported inconsistently [2]. In the NSWCR, AJCC-TNM data that are collected for clinical use are consolidated into summary case-level TNM-SG for epidemiological use. A summary case-level TNM-SG represents the highest TNM-SG at diagnosis (defined as within 120 days of date of diagnosis).

In Australia, AJCC-SG is currently not collected and reported at the national level. To determine the feasibility of collecting stage data for national reporting, Cancer Australia initiated a project whereby PBCRs would derive a stage surrogate aimed at providing the best estimate of AJCC-SG at diagnosis for the purpose of population-based analysis [5]. Referred to as Registry-Derived Stage (RD-stage), the stage group at diagnosis reflects T, N, and M values obtained from notification sources routinely available to PBCRs and derived by applying simplified AJCC business rules and algorithms developed by the Victorian Cancer Registry (VicCR).

In 2017, all Australian PBCRs, including the New South Wales Cancer Registry (NSWCR), derived RD-stage for five high incidence cancers diagnosed in 2011 – prostate, colorectal, breast, and lung cancer, and melanoma – and near-complete national cancer staging information was obtained.

Because the project required substantial manual effort and training for registry coders to obtain T, N, and M values from notification sources, as well as resources for application development, testing, and implementation of the business rules, the NSWCR determined the need to evaluate the value of collecting population-based RD-stage in addition to recording Degree of Spread (DoS), a stage surrogate that has been routinely collected by NSWCR for all non-haematopoietic cancers, where possible, since 1972. Numerical values for localised, regional and metastatic disease can be assigned relatively easily by registry and hospital coders and DoS has been extensively used for reporting and survival analysis by researchers and epidemiologists [2, 6–11].

The analyses aimed to identify: (i) for which of the five tumour groups does TNM stage (both AJCC-SG and RD-stage) provide more complete staging than the currently available summary stage (highest DoS at diagnosis), (ii) for which of the five tumour groups does RD-staging (compared to DoS) provide greater alignment with the more clinically relevant AJCC-SG, and (iii) which staging system is more appropriate for determining mortality and survival outcomes. Results of these analyses will contribute to a national discussion by state and territory PBCRs about which high incidence cancers national staging data should and can be annually collected and reported. It will also provide useful information for researchers and epidemiologists interested in using stage data from PBCRs.

## Methods

### Description of the NSWCR system

Under the *NSW Public Health Act 2010*, there is a mandatory requirement to report notifiable cancer cases to NSWCR by facilities that diagnose, manage, or treat cancer patients – these include public and private hospitals, public and private pathology laboratories, private and public day procedure centres, cancer treatment facilities, and residential aged care facilities [12]. Death data are notified from the NSW Registry of Births, Deaths and Marriages. Coded death data are supplied by the Australian Bureau of Statistics.

Notification data which pertains to a unique cancer type for the patient are consolidated to an incident cancer case through a process that entails computer-embedded business rules (implemented in the registry system) and manual coding.

### Description of staging data in the NSWCR system

#### AJCC-TNM stage data

AJCC stage, which is a non-mandatory data item, has been inconsistently collected at a local health district level from 2006 onwards from clinical and pathological documentation only and are not available for each cancer case, nor at a population level. In the NSWCR, AJCC stage data are stored in 11 data fields which describe: clinical and pathological component T, N, and M values; staging date; staging timing (whether or not staging occurred at time of diagnosis, defined as within 120 days from date of diagnosis); AJCC-SG; the clinical or pathological basis for AJCC-SG; and the edition of the AJCC staging manual used to determine stage. The AJCC-SG is automatically derived from T, N, and M values by business rules based on AJCC-TNM algorithms with the exception of prostate cancer, where Gleason score and prostate specific antigen (PSA) are not factored into the algorithms, resulting in a simplification of the AJCC-SG [4].

In the NSWCR, AJCC-SG data derived from notification sources for clinical use are consolidated into summary AJCC-SG for epidemiological use. The consolidation of T, N, and M values from multiple notification sources into a single cancer case reflects a hierarchy: the higher T, N, and M value (within 120 days) will override the lower value.

#### DoS stage data

DoS stage is also non-mandatory for collection, however has been recorded in the NSWCR, where applicable and available, at a population-based level for all non-haematopoietic cancers since 1972, and reflects the extent of disease at diagnosis. Tumours are categorised into four groups – in-situ, localised, regional, and distant – as defined by the International Agency for Research on Cancer (IARC) [13] (see Additional file 1: Table S1).

Although hospitals originally provided DoS manually, it is now provided in electronic coded notifications where it reflects information available in medical records. When multiple notifications are resolved to a single case in NSWCR, computer-embedded business rules determine the DoS at diagnosis (summary stage) based on the highest DoS within 120 days of the date of diagnosis, whether that DoS is from an inpatient electronic notification or assigned by a NSWCR coder based on a pathology report.

### Description of the RD-staging project

RD-stage consists of numerical stages I-IV. The aim of the RD-staging project was to provide population-based data for five high incidence tumour groups (prostate, colorectal, breast, lung, and melanoma) diagnosed in 2011. The NSWCR only staged eligible invasive cancers with a morphology code ending in /3, in-situ tumours were excluded (see Additional file 1: Table S2 for eligible tumour morphology and topography codes). Cases deemed ineligible included: (i) sarcomas and lymphomas of the breast, colon, rectum, lung, and prostate; (ii) carcinoid tumours of the colon and rectum; and (iii) transitional cell carcinomas of the prostate. Business rules to derive RD-stage using T, N, and M values were provided to the NSWCR by VicCR. For eligible cases where T, N, and M values were not already recorded, values were assigned using all routinely available notification sources in NSWCR within 120 days of diagnosis – these included scanned pathology reports, electronic hospital notifications (using DoS data and ICD10 metastatic codes), and clinical information (if available). Values were assigned by registry coders supervised by a pathologist using the AJCC 7th Edition [4]. For prostate cases, business rules also incorporated PSA and Gleason scores. Where RD-stage could not be derived due to incomplete information provided by T, N, and M, RD-stage was recorded as *stage missing* or *stage not-applicable*.

**Fig. 1:**
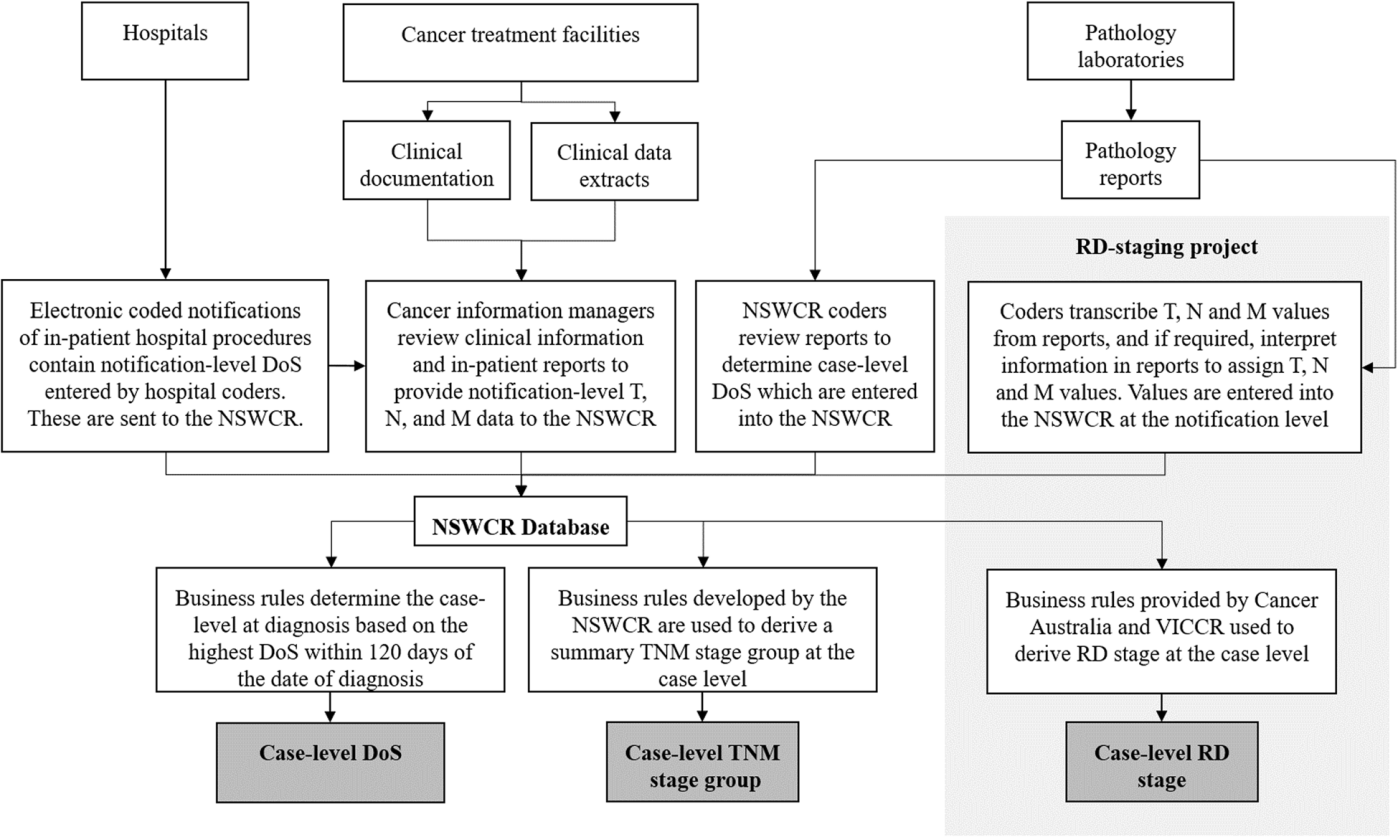
A summary of how AJCC-TNM, RD-stage, and DoS staging data was obtained. The procedures described in the grey box were performed as part of the RD-staging project and was not part of routine data collection procedures

Fig. 1 provides further information for how stage data was obtained.

### Evaluating completeness of stage data

To determine for which of the five cancers TNM stage data (both AJCC-SG and RD-stage) provided more complete staging than the current conventional DoS summary stage, stage data were extracted from NSWCR (pre- and post- RD-staging project) and compared across (i) tumour groups, (ii) stage systems, and (iii) time periods.

### Evaluating alignment across staging systems

We compared alignments between surrogate stage variables (DoS/RD-stage) with AJCC-SG. Expected mappings were developed from inspecting (i) RD-staging business rules provided by VicCR, (ii) NSWCR business rules for deriving AJCC-SG, (iii) NSWCR business rules for consolidating notification data for DoS and, (iv) IARC documentation. To confirm our expected mappings, we compared expected and observed cross-tabulated frequency distributions using a subset of NSWCR data. All staging information available for eligible RD-staging cases were extracted for analysis. For each tumour group, overall agreement measures (concordance and kappa) were calculated. Sensitivity and specificity were calculated for each stage grouping, similar to Kwan and colleagues [14]. To ensure staging data were comparable, all DoS 7 (invasion of adjacent organs and regional lymph nodes involved) were re-assigned to DoS 3 (regional lymph nodes) (see Additional file 1: Table S1). Pathologically and clinically staged AJCC-SG were consolidated (prioritising pathologically staged values where possible), and AJCC-SGs were collapsed from alpha-numerical to numerical classifications (stage I, II, III or IV).

### Evaluating which staging system is more appropriate for determining survival outcomes

In epidemiological analyses, stage data are most commonly used as a prognostic factor to estimate outcomes, particularly mortality and survival. We compared survival models developed from each of the three stage variables (RD-stage, AJCC-SG, or DoS). For lung and colorectal cancer cases we fitted Cox proportional hazards regression models to compare the relative contribution of RD-stage, AJCC-SG, or DoS as an explanatory variable to survival time. Patient demographic information (age at diagnosis and sex) are readily available in the NSWCR, and were also considered as explanatory variables. Mortality follow-up data, including cancer-specific causes of death, were available within the NSWCR up until the end of 2014. Prostate, breast and melanoma cancers generally have high 5-year survival rates (ranging from 90.6 to 95% based on Australian cancer data, 2010–2014) compared to lung and colorectal cancers (lung cancer 5-year survival in men and women in 2010–2014 are 14.5 and 19.6% respectively, whereas colorectal survival rates in men and women are 69.0 and 70.0%) [15]. Only lung and colorectal cancer were chosen for the 4-year survival analyses as we expected higher numbers of deaths to occur in these patients compared to prostate, breast and melanoma. We checked the proportional hazards assumption using Schoenfield residuals, and transformed variables as appropriate. Stage and age variables were found to violate the proportional hazards assumption. Stage variables were stratified. Transformation of age at diagnosis to a categorical variable (using age group categories described in Benitez-Majano and colleagues [3]) minimised violation of the proportional hazards assumption.

We additionally fitted logistic regression models to compare the relative contribution of the association between RD-stage, AJCC-SG, or DoS and all-cause mortality after a 1-year period from date of diagnosis. We examined Akaike information criterion (AIC) and Akaike weights to compare across each set of models. All calculations and visualisations were performed in R statistical software version 3.4.1.

## Results

A total of 25,299 NSW cases were identified as eligible for RD staging as of 15 June 2018 and extracted from the NSWCR for analysis. These included 3890 melanoma cases, 7223 prostate cases, 4770 colorectal cases, 4798 breast cases and 3618 lung cases (Table 1). Of these we found 1860 cases could not be RD staged due to missing information (*N* = 1142) or staging was non-applicable (*N* = 718). There were 2097 cases without a TNM stage due to missing information (*N* = 2071) or staging was non-applicable (*N* = 26). There were 3280 cases without a DoS value due to missing information (*N* = 3175), or staging was non-applicable (*N* = 105).

**Table 1:** NSWCR staging data completeness* pre- and post- RD-staging for melanoma, prostate, colorectal, breast, and lung cancer cases diagnosed in 2011

Tumour group	Pre-RD-staging ^a^			Post-RD-staging ^b^			
	AJCC-SG staged cases (n,%)	DoS staged cases (n,%)	Total number of cases in NSWCR	AJCC-SG staged cases (n,%)	DoS staged cases (n,%)	RD-staged cases (n,%)	Total number of cases eligible for RD-staging
Melanoma	367 (8.78%)	4019 (96.19%)	4179	3804 (97.79%)	3791 (97.48%)	3801 (98.68%)	3890
Prostate	1737 (22.53%)	5449 (71.58%)	7710	6946 (96.17%)	5147 (72.16%)	6919 (98.62%)	7223
Colorectal	2620 (50.94%)	4726 (92.07%)	5143	4217 (88.41%)	4460 (93.7%)	4244 (91.29%)	4770
Breast	3688 (53.13%)	6450 (92.93%)	5155	4457 (92.89%)	4549 (94.81%)	4518 (96.66%)	4798
Lung	2078 (55.05%)	3180 (83.91%)	3794	2778 (77.34%)	3072 (85%)	2957 (87.23%)	3618
Total	10,490 (37.8%)	23,824 (86.15%)	25,981	22,202 (91.47%)	21,019 (86.88%)	22,439 (95.16%)	24,299

* Non-applicable cases were excluded from analyses

^a^ Data extracted from NSWCR at 23 June 2017

^b^ Data extracted from NSWCR at 15 June 2018

### Evaluating completeness of stage data

Prior to undertaking the RD-staging project (June 2017), AJCC-SG data were available for less than half of eligible cases (see Table 1). In June 2018, as a result of the staging project, AJCC-SG and RD-stage were available for over 90% of eligible cases. RD-staging improved completeness for TNM derivations (AJCC-SG/RD-stage) across all stage groups, with melanoma showing the greatest change in completeness followed by prostate, breast, colorectal, and lung cancer. Prostate cancer had the lowest staging completeness for DoS pre- and post- RD-staging, followed by lung cancer.

### Evaluating alignment across staging systems

**Fig. 2:**
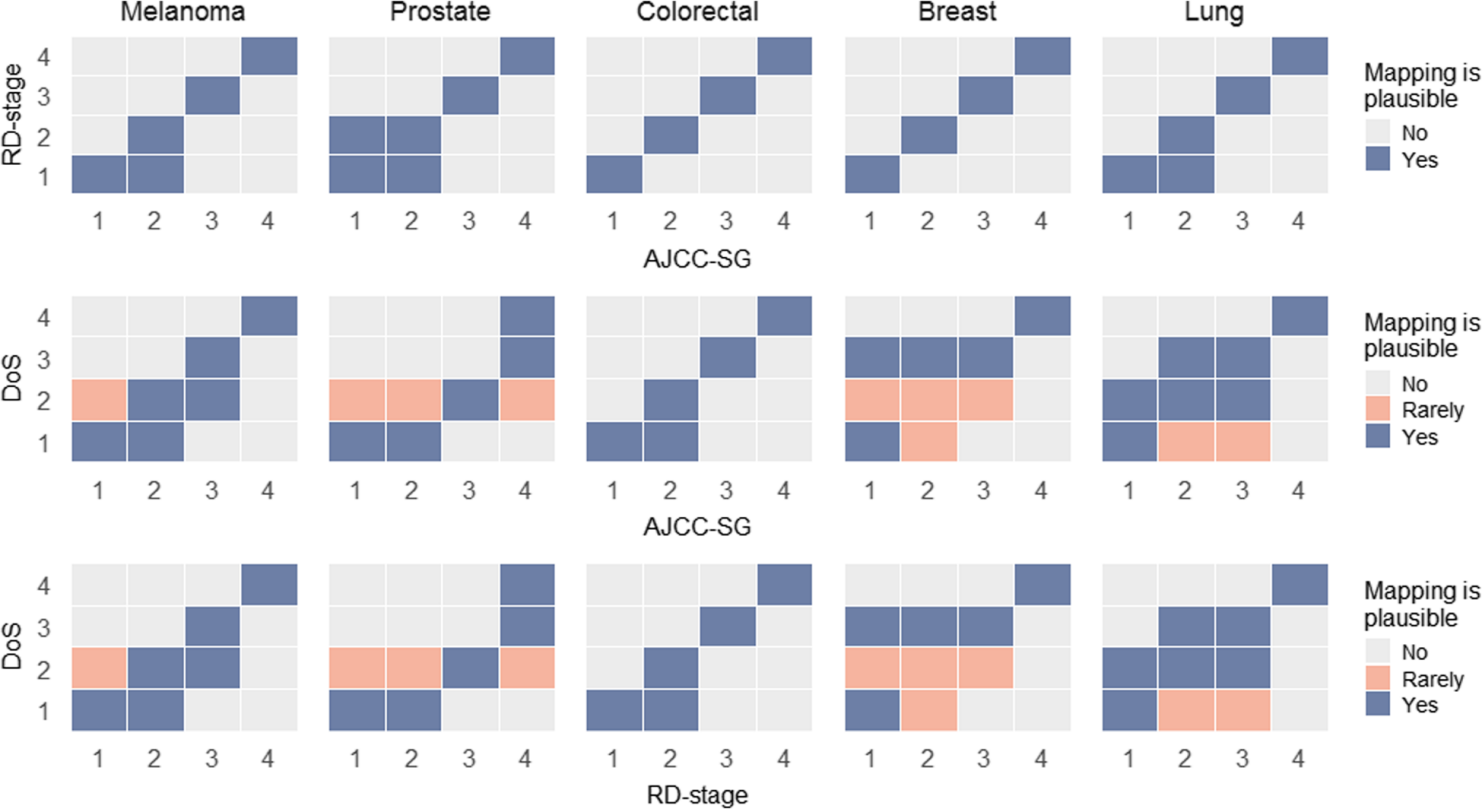
Expected distributions of cases based on mappings. The top and middle panel show the expected cross-tabulated case distributions for each AJCC-SG by RD-stage (top) and DoS (middle). The bottom panel shows the expected cross-tabulated case distributions for each RD-stage by DoS

**Fig. 3:**
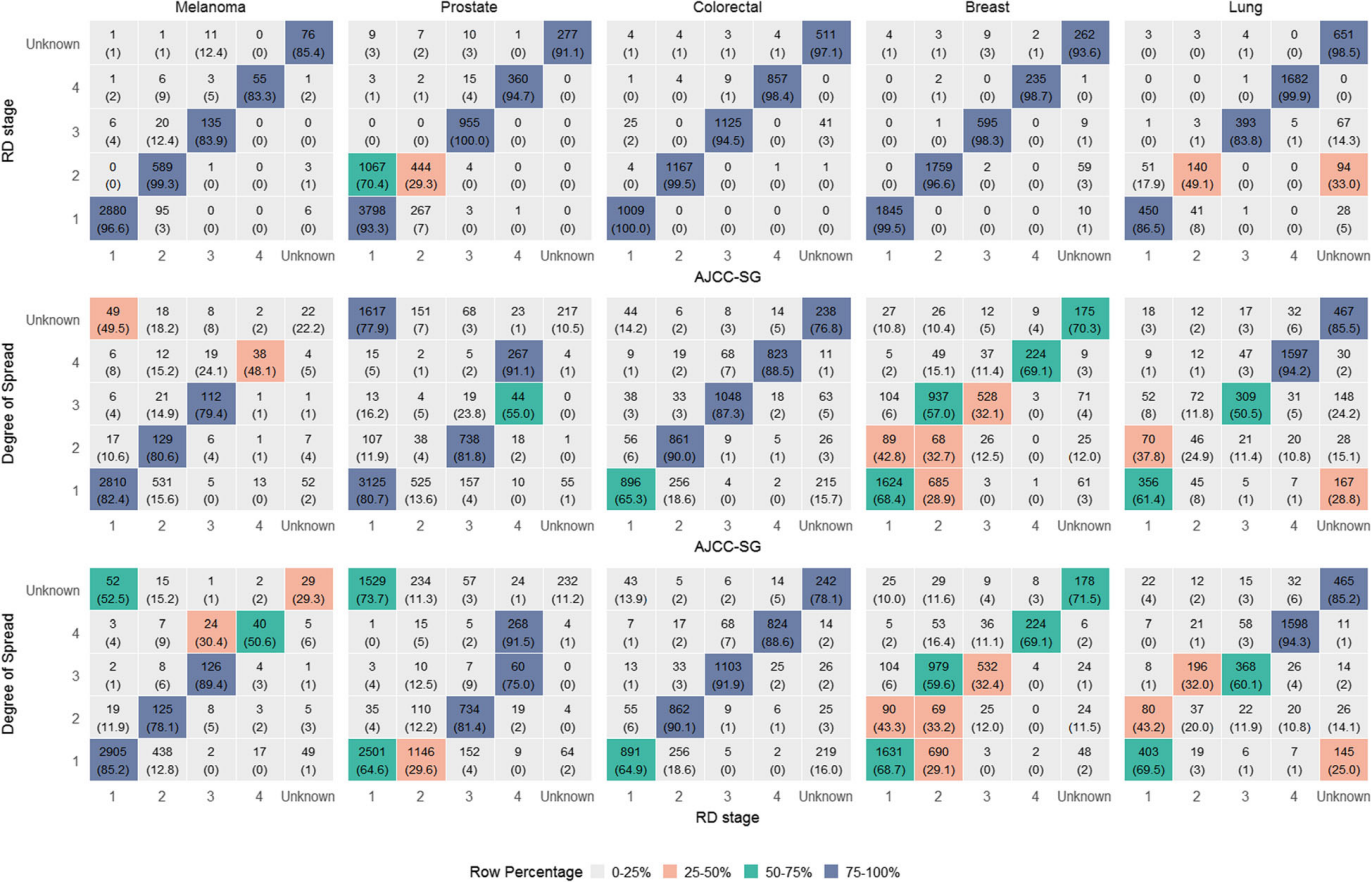
Frequency distribution of all eligible cases. The top and middle panel show the number of cases and row percentage across each AJCC-SG by RD-stage (top) and DoS (middle). The bottom panel shows the number of cases and row percentage across each RD-stage by DoS

Mappings were developed across all three staging systems based on examination of relevant stage system documentation (details provided in Table 2 and Additional file 1: Tables S3, S4, S5, S6 and S7). Mappings (see Fig. 2) showed RD-stage to AJCC-SG alignments were mostly linear (RD-stage I = AJCC-SG I, RD-stage II = AJCC-SG II, etc.), whereas comparability of DoS and AJCC-SG was less well-defined with the exception of the colorectal tumour group. These findings were also reflected in our analysis of NSWCR case data where higher agreement scores were more evident in comparisons of AJCC-SG/RD-stage compared to AJCC-SG/DoS (see Fig. 3 and Additional file 1: Table S8).

**Table 2:** Explaining non-linear stage group mappings between the three staging systems

Tumour group	Mapping details
Melanoma	- T2b N0 M0 derives to AJCC-SG II and, by simplified business rules which do not substage, to RD-stage I. - Any T with N0 M0 maps to DoS 1 (rarely 2) and either a RD-stage/AJCC-SG I or II depending on the T value assigned. - In NSWCR, DoS 2 (in the absence of regional lymph node metastasis) has conventionally been assigned to: (i) a primary cutaneous melanoma involving subcutaneous fat (Clark’s level V) which could potentially map to AJCC-SG/RD-stage I or II (most likely II) and (ii) a primary cutaneous melanoma with satellite nodules/in-transit nodules, which equates to N2c in AJCC staging (pathological AJCC-SG IIIBor IIIC and RD-stage III).
Prostate	- PSA and Gleason scores are not factored into the algorithms for deriving AJCC-SG in NSWCR. - VicCR business rules assign RD-stage I for cases either (i) without a PSA or Gleason score or (ii) both PSA < 10 and Gleason score ≤ 6. RD-stage II is assigned for cases where (i) PSA ≥10 or (ii) Gleason score > 7. Given the poor availability of PSA data in PBCRs generally, there is a tendency for down-staging of prostate cancer in NSWCR by both AJCC and RD-staging systems. - In NSWCR, a DoS cannot be assigned by a coder based on a core biopsy or transurethral resection of the prostate (TURP) unless there is a clear description of extraprostatic extension, in which case DoS 2 can be assigned. However, a DoS may be recorded in an associated electronic notification. This compares to AJCC-TNM and RD-staging, in which prostate cancer in a core biopsy or TURP alone can be assigned a T value and allocated to stages I or II, depending on the PSA and/or Gleason score. - In NSWCR, where PSA and/or Gleason score are unknown, a core biopsy diagnosis of prostate cancer would derive to AJCC-SG/RD-stage I. - In NSWCR, DoS 1 can be assigned when a prostatectomy shows cancer localised to the prostate; these cases correspond to T2 tumours = AJCC-SG and RD-stage II (and occasionally I). - In NSWCR, the majority of cases with DoS 2 would reflect cases for which a prostatectomy was performed and there was evidence of extraprostatic extension; these cases correspond to T3 tumours (AJCC-SG and RD-stage III). - Cases staged as T4 N0 M0 equate to DoS 2 but AJCC-SG/RD-stage IV. - Cases staged as any T with N1 M0 equate to DoS 3 in NSWCR, but AJCC-SG/RD-stage IV.
Colorectal	- Colorectal tumour extending beyond the muscle coat into subserosa only is assigned DoS 1, whereas these would likely be staged as pT3 (AJCC-SG/RD-stage II).
Breast	- An invasive tumour of any size localised to the breast would be assigned DoS 1. - DoS 2 would be assigned by a coder if there was skin, nipple (associated Paget disease), or chest wall involvement (effectively T4 tumours). - Any lymph node involvement other than isolated tumour cells alone is assigned DoS 3.
Lung	- Tumours staged as T2b N0 M0 (AJCC-SG IIA) would simplify to RD-stage I as the VicCR business rules do not substage T2 tumours. - Lung tumours that invade pleura or immediate adjacent tissues or organs are assigned DoS 2 by NSWCR coders irrespective of tumour size, so a DoS 2 tumour could be equivalent to a T1-T4 tumour in AJCC-TNM staging. Therefore, in the absence of regional lymph node involvement, these tumours could be staged as AJCC-SG/RD-stage I, II, or III. - The presence of a malignant pleural effusion has been variably interpreted by NSWCR and hospital coders as DoS 2 or DoS 4, although mainly as DoS 4, which equates to M1a (AJCC and RD-stage IV).

Compared to DoS, RD-staging provided greatest improvements in accuracy (in terms of alignment to AJCC-SG) for prostate and breast cancers followed by lung cancer and melanoma. For colorectal cancer, we found all three classification systems showed near-linear alignment with scores of > 80% for all accuracy scores.

### Evaluating which staging system is more appropriate for determining survival outcomes for lung and colorectal cancers

From examining AIC values (see Additional file 1: Tables S9 and S10), models using AJCC-SG consistently showed the highest fit and models using DoS consistently showed the poorest fit. Akaike weights indicated models using AJCC-SG best explained survival outcomes. In 4-year multivariable Cox proportional hazards models (see Additional file 1: Table S10), across all model sets, we observed highly similar hazard ratios (HRs) among RD-stage, AJCC-SG, and DoS models, suggesting that DoS and RD-stage are adequate alternatives to AJCC-SG for estimating survival and mortality outcomes for colorectal and lung cancers. From examining an additional set of 4-year multivariable Cox proportional hazards models where stage groups were not stratified, we observed models using DoS underestimated HRs for higher stage groups. Similar trends were found in 1-year all-cause mortality logistic regression models (see Additional file 1: Table S9) and univariable 4-year Cox proportional hazards models (see Additional file 1: Table S11).

## Discussion

The NSWCR has implemented a range of innovative collection and processing applications to provide high-quality data for standard cancer registry fields, as well as collection of both DoS and AJCC-TNM data where possible. As such, the NSWCR was uniquely placed to compare the three staging systems. For the five cancers with the highest incidence in Australia, we compared completeness of stage data between the three staging systems, and compared the alignment of RD-stage and DoS to AJCC-SG. We provide a discussion of the comparability of the staging systems for each individual tumour group.

### Prostate cancer

Overall, we found RD-staging (compared to DoS) provided greater stage data completeness and accuracy (alignment to AJCC-TNM) for prostate cancer cases. RD-staging provided stage data for 98% of prostate cases compared to only 72% for DoS. Previous NSWCR studies have similarly shown low DoS stage data completeness for prostate cancer [16, 17]. RD-stage data was also much more aligned to AJCC-TNM with concordance/kappa scores of 80%/64%, compared to only 68%/35% for DoS. Based on clear improvements in stage data completeness and accuracy compared to DoS, prostate cancer would be a clear candidate for RD-staging in the NSWCR.

It is important to note the caveats that apply to both RD-staging and NSWCR’s AJCC staging systems. It is expected that the NSWCR will have a higher number of AJCC-SG I prostate cases that are actually AJCC-SG II given PSA and Gleason score are not factored into the business rule algorithm to calculate AJCC-SG. This was also reflected in the RD-stage where these non-anatomic variable were unavailable – in our study sample of 7223 prostate cases we found only 31% (*N* = 2210) had a valid Gleason score and only 23% (*N* = 1626) had a valid PSA score. Overall, this means that both AJCC-SG and RD-stage will potentially underestimate the incidence of stage group II prostate cancers as both NSWCR and VICCR algorithms simplify them to stage group I. With these points considered, within the NSWCR, RD-staging (rather than AJCC-SG) will provide stage group classifications that would more closely align to current (7th and 8th) AJCC editions for prostate cancer.

### Colorectal cancer

For colorectal cases, we found summary stage from DoS an adequate surrogate staging system: compared to DoS, RD-staging did not improve stage data completeness, and RD-stage only provided a small amount of improvement in accuracy (with concordance/kappa scores of 99%/99 and 88%/83% for RD-stage and DoS respectively). Both DoS and RD-stage 4-year multivariable Cox proportional hazards survival models showed highly similar hazard ratios (HR) to more clinically relevant AJCC-SG models when the stage variable was stratified, which suggest both stage variables are suitable alternatives to AJCC-SG for survival modelling. However, we observed DoS consistently underestimated odds ratios (ORs) and HRs in 1-year all-cause mortality logistic regression models, univariable 4-year Cox models, and multivariable 4-year Cox models.

### Melanoma

For melanoma, we found concordance/kappa scores were higher for RD-stage (97%/91%) compared to DoS (83%/44%). However, compared to DoS, RD-staging provided only minimal improvements in terms of stage data completeness (DoS was available for 98% of melanoma cases compared to 99% for RD-stage).

### Breast cancer

For breast cancer, RD-staging showed near-perfect alignment to more clinically relevant AJCC-SG (with very high concordance/kappa scores of 100%/100% for RD-stage and fairly low scores of 56%/38% for DoS). However, compared to DoS, RD-staging provided minimal improvements in terms of stage data completeness (DoS was available for 95% of breast cancer cases compared to 97% for RD-stage).

### Lung cancer

We found RD-staging provided small improvements in stage data completeness (DoS was available for 85% of lung cancer cases compared to 87% for RD-stage). RD-staging however showed improvements in alignment to AJCC-SG (concordance/kappa scores increased moderately from 86%/74% for DoS to 96%/93% for RD-stage). As seen with colorectal cancer, lung cancer multivariable 4-year Cox proportional hazards survival models showed similar HRs among RD-stage, AJCC-SG, and DoS models, however this was only seen when the stage variable was stratified.

### RD-staging in the NSWCR – procedure and workload compared to AJCC-TNM and DoS

Both RD-stage and AJCC-SG data were derived from T, N and M values which were sourced from manual review of pathology reports by NSWCR coders (as part of the RD-staging project), and/or manual review of hospital in-patient notification and other clinical information sources by Cancer Information Managers (CIMs) (as part of routine NSWCR data collection). The RD-staging project, conducted in 2017, involved manual collection of T, N and M values from pathology reports of melanoma, breast, prostate, colorectal and lung cancer cases diagnosed in 2011. This exercise not only provided the RD-stage data, but also resulted in a substantial increase in AJCC-SG data coverage. While a formal comparison of procedure and workload for RD-staging and AJCC-TNM staging cannot be performed, we can provide comments around (i) routine data collections and (ii) data collections performed specifically for the RD-staging project.

Routine TNM data collections in the NSWCR are performed by CIMs and involve transcribing data from reports held in either data extracts from cancer treatment centres, or reports held at point of care in the NSWCR. Complete population coverage is not possible as CIMs generally collect data from public (as opposed to private) hospitals and treatment centres. When there are data inconsistencies or when data is missing, CIMs review clinical documents from cancer treatment centres and all inpatient hospital notifications sourced from hospitals. This can take years to get through full review due to the volume of inpatient notifications generated. The proportion of missing data is variable, however generally data completeness is poor across the board. It is also worth noting that even when the CIMs manually review 100% of patients in a period, recovering and providing TNM values for 100% of those patients is not possible primarily due to data governance (e.g. private consult notes cannot be provided and public treatment referral letters miss key information), and also due to TNM not being essential to some treatment decisions in some treatment modalities and/or protocols.

Collection of DoS is conducted routinely within the NSWCR and is part of coding a cancer case. DoS collection adheres to published IARC categories [13] and is comparatively straightforward for the tumours staged in this study. Generally, there is higher stage data completeness for DoS compared to collection of T, N and M data.

The RD-staging project involved extensive training of NSWCR coders to recognise and assign T, N and M based on review of available pathology reports in the NSWCR. Where T, N and M values were not able to be transcribed – information in reports were reviewed and interpreted by coders to assign T, N and M. We estimated NSWCR coders completed manual TNM staging of 16,007 cases within 61 working days. The time spent on the RD-staging project however impacted on routine coding procedures – for other PBCRs where additional resourcing is not available, collection of stage data may not be worthwhile.

### Stage data collection in PBCRs – future directions

Stage is currently not considered an essential variable for reporting by the International Association of Cancer Registries (IACR). However, with growth in capacity for PBCRs to store and manage clinical data, collection of stage data is becoming more feasible [4]. Furthermore, there is increasing interest in measuring global cancer survival outcomes [2, 18, 19]. Growing interest in using stage data for clinically-oriented population studies has also created a need for collection of TNM stage data.

While AJCC-TNM data are not mandatory for collection, a 2013 comparative analysis of international PBCRs found AJCC-TNM stage data were collected from PBCRs in 10 of 12 jurisdictions [2]. In England, increasing stage data completeness has been a national priority in recent years and resources have been specifically allocated to improve data collection processes [20]. In the United States, AJCC-TNM stage data have been collected since 2004 under a national Collaborative Stage Data Collection System which has recently been expanded to incorporate information on related biomarkers and prognostic factors [4]. Other PBCRs have conducted and published evaluations of completeness and accuracy of AJCC-TNM stage data within their respective registries [21–23]. Australian PBCRs are considered well-resourced, high quality PBCRs [2, 24] and accordingly, should aim to meet high standards in cancer reporting, including provision of complete and accurate AJCC-TNM stage data.

A limitation of RD-staging is that other countries are not familiar with RD-stage and have no access to TNM information necessary for RD stage. In 2018 the Union for International Cancer Control (UICC) released *Essential TNM* a process for collecting stage data in PBCRs in low and middle income countries where there are insufficient resources to derive complete TNM data. [25] *Essential TNM* is aligned with the UICC staging system, not AJCC –differences between the two systems have previously been documented [26]. While a comprehensive formal mapping of *Essential TNM* to AJCC TNM and DoS was outside the scope of this study, we provide some brief comments based on the Essential TNM User Guide. [27] Generally, *Essential TNM* aligns more closely with DoS: DoS 1 (and DoS 6) would equate to L1/L2, DoS 2 would equate to A1/A2, DoS 3 and 7 map to R+, and DoS 4 map to M+. Examining the staging of prostate cancer in more detail – *Essential TNM*, like DoS, defaults N+ tumours to Stage III, which we found to map across AJCC SGs III and IV. T4 N0 M0 also maps to AJCC SG IV but aligns with DoS 2 and would align with *Essential TNM* TA (locally advanced). Given the simplification of T staging and the assumption of Stage III disease for node-positive prostate cancer, DoS and *Essential TNM* are likely to align in under-staging AJCC-SG IV cancers as well as resulting in a higher number of unknown stage cases for biopsy-only cases. It would be reasonable to consider DoS as a staging system for PBCRs in low and middle income countries given there is documentation available for most tumour groups (not just breast, cervix, colon and prostate cancer) [13].

Our comparisons of survival models show DoS in the context of a PBCR remains useful for epidemiological studies as traditionally intended and used. In this paper we provide comprehensive DoS to AJCC-TNM mappings based on the 7th edition AJCC which will be useful for researchers interested in consolidating stage data across the different stage classification systems. DoS can potentially be used in conjunction with TNM-derived data through mapping algorithms, as explored in previous studies [2, 14]. [28]

At its meeting in November 2018, the Australasian Association of Cancer Registries (AACR) discussed the value and feasibility of prospectively collecting and providing national stage data. In light of the findings of our analysis and those provided by a similar analysis undertaken by the South Australia Cancer Registry, there was a preliminary agreement for Australasian PBCRs to consider prospective collection of stage data, where possible, for melanoma, breast, and colon cancers with a diagnosis date of 2017 onwards. Lung cancers are considered difficult to accurately stage based on information available to PBCRs, and comprehensive AJCC-TNM stage data are already collected by the state-based Prostate Clinical Cancer Registries.

In light of the move toward Structured Reporting of Cancer nationally and internationally, the The Royal College of Pathologists of Australasia (RCPA) has issued a Position Statement [29] advising its Fellows to implement AJCC Staging (8th edition). In general, Australian pathologists have historically used AJCC staging in practice and NSWCR implemented Business Rules for AJCC accordingly.

### Study limitations

We acknowledge that the findings drawn from this study may not be the same across other cancer registries, or for other diagnosis years or tumour groups. Our analyses only used data from the NSWCR for a subset of melanoma, prostate, colorectal, breast, and lung cancer cases diagnosed in 2011 that were eligible for RD-staging. Other Australian cancer registries will have different stage data collection practices – the value of RD-staging within their respective registry may be determined by different factors. Additionally, survival analyses conducted in this study only examined outcomes at 1 and 4 years after diagnosis, whereas in practice, models typically examine survival at 5 or 10 years after diagnosis.

## Conclusion

Complete and accurate stage information is important for use in epidemiological analyses which inform cancer prevention and control policies, programs, and treatment decisions. These analyses provides an evidence-based approach that can be used to inform decision-making for resource planning and potential implementation of RD-staging in PBCRs. For each of the five high incidence cancers included in this study, we compared the level of improvements RD-staging provided in terms of completeness and accuracy (alignment to more clinically relevant AJCC-TNM) over DoS. RD-staging may assist other PBCRs to record stage aligned with AJCC-TNM. We found RD-staging provided greatest completeness and alignment scores for prostate cancers followed by breast, then melanoma and lung cancers. For colorectal cases, summary stage DoS has been shown to be an adequate surrogate staging system. While a TNM-based staging system would be preferable, simplified staging systems such as DoS and *Essential TNM* may suffice for certain tumour groups or where PBCR resources and notifications are lacking.

## Additional file

Additional file 1: **Table S1.** DoS summary stage IARC definitions [13] **Table S2.** ICD-O3 histology and topography codes eligible for staging under the AJCC-TNM staging classification. **Table S3.** Melanoma. **Table S4.** Prostate. **Table S5.** Colorectal. **Table S6.** Breast. **Table S7.** Lung. **Table S8.** Test characteristics* comparing RD-stage and DoS to AJCC-SG stage for each tumour group and stage. **Table S9.** Summary of 1-year all-cause mortality logistic regression models by cancer and staging system. **Table S10.** Summary of multivariable 4-year Cox proportional hazards survival models by cancer and staging system. **Table S11.** Summary of univariable 4-year Cox proportional hazards survival models by cancer and staging system. (PDF 547 kb)

## Data Availability

Permissions were obtained from the Cancer Institute NSW and Cancer Australia for permission to use NSWCR data for this analysis. NSWCR data, including the stage data used in this study, can be accessed via the NSWCR website https://www.cancer.nsw.gov.au/data-research/access-our-data.

## Abbreviations

AIC: Akaike information criterion
AJCC: American Joint Committee on Cancer
AJCC-SG: American Joint Committee on Cancer Stage Group
AJCC-TNM: American Joint Committee on Cancer Tumour-Node-Metastasis
DoS: Degree of Spread
HR: Hazard ratio
IARC: International Agency for Research on Cancer
NSW: New South Wales
NSWCR: New South Wales Cancer Registry
OR: Odds ratio
PSA: Prostate-Specific Antigen Test
RD-stage: Registry-Derived stage
TNM: Tumour-Node-Metastasis
TNM-SG: Tumour-Node-Metastasis Stage Group
UICC: Union for International Cancer Control
VICCR: Victorian Cancer Registry
