# Supplementary material for: Assessing a modified-AJCC TNM staging system in the New South Wales Cancer Registry, Australia
Source: BMC Cancer. 2019 Aug 28;19:850. doi: 10.1186/s12885-019-6062-x (PMC6714314; doi:10.1186/s12885-019-6062-x)
Supplement: Supplementary file 1 — Table S1. DoS summary stage IARC definitions [13] Table S2. ICD-O3 histology and topography codes eligible for staging under the AJCC-TNM staging classification. Table S3. Melanoma. Table S4. Prostate. Table S5. Colorectal. Table S6. Breast. Table S7. Lung. Table S8. Test characteristics* comparing RD-stage and DoS to AJCC-SG stage for each tumour group and stage. Table S9. Summary of 1-year all-cause mortality logistic regression models by cancer and staging system. Table S10. Summary of multivariable 4-year Cox proportional hazards survival models by cancer and staging system. Table S11. Summary of univariable 4-year Cox proportional hazards survival models by cancer and staging system. (PDF 547 kb) [file 12885_2019_6062_MOESM1_ESM.pdf]

Appendix

Table S1. DoS summary stage IARC definitions [13]

Definition	Description	Corresponding DoS code in NSWCR*
Localised	i. Invasive cancer confined to organ or tissue of origin<br>ii. Intraluminal extension where specified	1 – Localised to the tissue of origin
Regional	i. By direct extension to adjacent organs/tissues<br>ii. To regional lymph nodes<br>iii. By direct extension and lymph node involvement	2 – Invasion of adjacent tissue or organs
		3 - Regional lymph nodes
		7 - Invasion of adjacent organs and regional lymph nodes involved
Distant	i. Direct continuity to organs other than above<br>ii. Discontinuous metastasis<br>iii. To distant (non-regional) lymph nodes	4 - Distant metastases
In-situ	Intraepithelial, non-invasive, non-infiltrating	6 - In-situ

* NSWCR DoS values were used for analyses.

Table S2. ICD-O3 histology and topography codes eligible for staging under the AJCC-TNM staging classification

	ICD-O3 histology codes	ICD-O3 topography codes
Melanoma	8720-8790	C440-C449; C510-C519; C600-C609; C632
Prostate	8000-8110; 8140-8576; 8940-8950; 8980-8981	C619
Colorectal	8000-8152; 8154-8231; 8243-8245; 8250-8576; 8940-8950; 8980-8981	C180; C182-C189; C199; C209
Breast	8000-8576; 8940-8950; 8980-8981; 9020	C500-C509
Lung	8000-8576; 8940-8950; 8980-8981	C340-C349

Table S3-7. Mapping unique combinations of T, N, M to RD-stage, AJCC-SG, and DoS

S3. Melanoma

T	N	M	RD-stage	AJCC-SG	DoS
1a	0	0	I	IA	1
1b	0	0	I	IB	1
2a	0	0	I	IB	1(2)
2b	0	0	I	IIA	1(2)
3a	0	0	II	IIA	1(2)
3b	0	0	II	IIB	1(2)
4a	0	0	II	IIB	1(2)
4b	0	0	II	IIC	1(2)
1 to 4a	1a	0	III	IIIA	3
1 to 4a	2a	0	III	IIIA	3
1 to 4b	1a	0	III	IIIB	3
1 to 4b	2a	0	III	IIIB	3
1 to 4a	1b	0	III	IIIB	3
1 to 4a	2b	0	III	IIIB	3
1 to 4a	2c	0	III	IIIB	2
1 to 4b	1b	0	III	IIIC	3
1 to 4b	2b	0	III	IIIC	3
1 to 4b	2c	0	III	IIIC	2
Any	3	0	III	IIIC	3
Any	Any	1	IV	IV	4

S4. Prostate

T	N	M	RD-stage[a,b]	AJCC-SG	DoS
1a to c	0	0	I or II	I	NULL, 9 or I (rarely 2 from a core biopsy)
2a	0	0	I or II	I	1
1a to c	0	0	I or II	IIA	NULL, 9 or I (rarely 2 from a core biopsy)
2a to b	0	0	I or II	IIA	1
2c	0	0	I or II	IIB	1
1 to 2c	0	0	I or II	IIB	1
3a to b	0	0	III	III	2
4	0	0	IV	IV	2
Any	1	0	IV	IV	3 (DoS 7 for T3 and T4 tumours)
Any	Any	1	IV	IV	4

[a] RD-stage I cases are those (i) without a PSA or Gleason score or (ii) both PSA <10 and Gleason score ≤6.
[b] RD-stage II cases are those with (i) PSA ≥10 or (ii) Gleason score >7.

S5. Colorectal

T	N	M	RD-stage	AJCC-SG	DoS
1	0	0	I	I	1
2	0	0	I	I	1
3	0	0	II	IIA	2 (1)
4a	0	0	II	IIB	2
4b	0	0	II	IIC	2
1 to 2	1 or 1c	0	III	IIIA	3
1	2a	0	III	IIIA	3
3 to 4a	1 or 1c	0	III	IIIB	3
2 to 3	2a	0	III	IIIB	3
1 to 2	2b	0	III	IIIB	3
4a	2a	0	III	IIIC	3
3 to 4a	2b	0	III	IIIC	3
4b	1 to 2	0	III	IIIC	3
Any	Any	1a	IV	IVA	4
Any	Any	1b	IV	IVB	4

S6. Breast

T	N	M	RD-stage	AJCC-SG	DoS
1mi/1	0	0	I	IA	1 (2)
0	1mi	0	I	IB	3
1	1mi	0	I	IB	3
0	1	0	II	IIA	3
1	1	0	II	IIA	3
2	1	0	II	IIA	3 or 7
2	1	0	II	IIB	3
3	0	0	II	IIB	1 or 2
0	2	0	III	IIIA	3
1	2	0	III	IIIA	3
2	2	0	III	IIIA	3 or 7
3	1	0	III	IIIA	3 or 7
3	2	0	III	IIIA	3 or 7
4	0	0	III	IIIB	2
4	1	0	III	IIIB	7
4	2	0	III	IIIB	7
Any	3	0	III	IIIC	3 or 7
Any	Any	1	IV	IV	4

S7. Lung

T	N	M	RD-stage	AJCC-SG	DoS
1a	0	0	I	IA	1
1b	0	0	I	IA	1
2a	0	0	I	IB	1 or 2
2b	0	0	I	IIA	1 or 2
1a	1	0	II	IIA	3
1b	1	0	II	IIA	3
2a	1	0	II	IIA	3
2b	1	0	II	IIB	3
3	0	0	II	IIB	1 or 2
1a	2	0	III	IIIA	3
1b	2	0	III	IIIA	3
2a	2	0	III	IIIA	3
2b	2	0	III	IIIA	3
3	1	0	III	IIIA	3
3	2	0	III	IIIA	3
4	0	0	III	IIIA	2 or 1
4	1	0	III	IIIA	3
1a	3	0	III	IIIB	3
1b	3	0	III	IIIB	3
2a	3	0	III	IIIB	3
2b	3	0	III	IIIB	3
3	3	0	III	IIIB	3
4	2	0	III	IIIB	3
4	3	0	III	IIIB	3
Any	Any	1a	IV	IV	4
Any	Any	1b	IV	IV	4

Table S8. Test characteristics* comparing RD-stage and DoS to AJCC-SG stage for each tumour group and stage

Tumour group	Stage group	AJCC-SG/RD-stage				AJCC-SG/DoS				RD-stage/DoS			
		C (%)	K (%)	SE (%)	SP (%)	C (%)	K (%)	SE (%)	SP (%)	C (%)	K (%)	SE (%)	SP (%)
Melanoma	I	96.5	90.6	99.8	89.5	82.9	43.7	99.0	38.2	85.7	49.6	99.2	43.0
	II			83.0	100.0			18.6	99.2			21.6	99.1
	III			97.1	99.3			78.9	99.2			78.8	99.6
	IV			100.0	99.7			71.7	99.0			62.5	99.1
Prostate	I	80.3	63.6	78.0	86.8	67.8	34.7	95.9	62.1	56.9	24.7	98.5	48.4
	II			62.3	82.7			6.7	80.9			8.6	79.2
	III			97.8	100.0			2.1	98.5			1	98.3
	IV			99.7	99.7			78.8	99.5			75.3	99.6
Colorectal	I	99.0	98.6	97.1	100.0	87.5	83.4	89.7	91.7	88.1	84.1	92.2	91.8
	II			99.7	99.8			73.7	97.7			73.8	97.7
	III			99.2	99.2			92.8	97.1			93.1	97.6
	IV			99.9	99.6			97.1	97.1			96.2	97.2
Breast	I	99.9	99.8	100.0	100.0	55.8	37.8	89.1	73.1	55.2	37.4	89.1	73.4
	II			99.8	99.9			4	95.7			4	95.7
	III			99.7	100.0			88.9	72.5			89.3	71.8
	IV			100.0	100.0			98.3	97.8			97.4	97.8
Lung	I	96.3	93.5	89.6	98.2	85.5	74.4	73.1	97.4	83.7	72.8	80.9	98.7
	II			76.1	98.0			26.3	95.6			13.6	95.3
	III			99.5	99.6			80.9	93.3			81.1	90.5
	IV			99.7	99.9			96.5	93.5			96.8	93.0

* Test characteristics included concordance (C), kappa (K), sensitivity (SE), and specificity (SP). A total of 22,119 cases had complete staging information where RD-stage and AJCC-SG were not missing or non-applicable and were used for calculating test characteristics to assess similarities between RD-stage and AJCC-SG stage. A total of 20,041 cases had complete staging information where DoS and AJCC-SG were not missing or non-applicable, and 20,305 cases had complete staging information where DoS and RD-stage not missing or non-applicable.

Table S9. Summary of 1-year all-cause mortality logistic regression models by cancer and staging system

	Lung cancer			Colorectal cancer		
	Model 1 (RD-stage)	Model 2 (AJCC-SG)	Model 3 (DoS)	Model 1 (RD-stage)	Model 2 (AJCC-SG)	Model 3 (DoS)
Stage[a]	OR (95% CI), P-value	OR (95% CI), P-value	OR (95% CI), P-value	OR (95% CI), P-value	OR (95% CI), P-value	OR (95% CI), P-value
II	3.74 (2.63-5.33), P <0.001	2.18 (1.44-3.32), P <0.001	1.99 (1.39-2.85), P <0.001	2.81 (1.79-4.41), P < 0.001	2.69 (1.74-4.17), P < 0.001	2.08 (1.48-2.93), P < 0.001
III	5.12 (3.74-7.02) , P <0.001	4.54 (3.28-6.28), P <0.001	2.11 (1.64-2.73), P <0.001	3.10 (1.99-4.83), P < 0.001	3.01 (1.95-4.65), P < 0.001	1.61 (1.14-2.27), P = 0.006
IV	20.25 (15.32-26.79), P <0.001	19.01 (14.39-25.11), P <0.001	11.03 (8.81-13.82), P <0.001	33.70 (22.33-50.87), P < 0.001	32.66 (21.92-48.66), P < 0.001	18.52 (13.86-24.73), P < 0.001
AIC (w)	3,293 (0)	3,046 (1)	3,528 (0)	2,666 (0)	2,647 (1)	2,931 (0)

[a] Reference category is Stage group I.

Table S10. Summary of multivariable 4-year Cox proportional hazards survival models by cancer and staging system

	Lung cancer			Colorectal cancer		
Variable	Model 1 (RD-stage)	Model 2 (AJCC-SG)	Model 3 (DoS)	Model 1 (RD-stage)	Model 2 (AJCC-SG)	Model 3 (DoS)
	HR (95% CI), P-value	HR (95% CI), P-value	HR (95% CI), P-value	HR (95% CI), P-value	HR (95% CI), P-value	HR (95% CI), P-value
Sex [a]	0.91 (0.84-1.00), P =0.043	0.91 (0.83-0.99), P =0.037	0.9 (0.82, 0.98), P =0.013	1.07 (0.95, 1.21), P =0.285	1.08 (0.95, 1.22), P =0.223	1.06 (0.94, 1.2), P =0.330
Age group [b]:						
45 - 54	1.42 (0.82-2.21), P =0.116	1.29 (0.83-2.01), P =0.255	1.42 (0.92, 2.19), P =0.111	0.93 (0.62, 1.41), P =0.739	0.92 (0.61, 1.39), P =0.703	0.88 (0.59, 1.33), P =0.551
55 - 64	1.72 (1.13-2.62), P =0.112	1.63 (1.07-2.48), P =0.024	1.72 (1.14-2.59), P =0.010	0.97 (0.66, 1.41), P =0.857	0.98 (0.67, 1.43), P =0.920	0.89 (0.62, 1.3), P=0.555
65 - 74	1.92 (1.26-2.90), P =0.002	1.78 (1.18-2.70), P=0.007	1.90 (1.27-2.86), P =0.002	1.56 (1.08, 2.24), P =0.016	1.57 (1.09, 2.26), P =0.014	1.28 (0.90, 1.83), P=0.173
>75	2.67 (1.76-4.04), P <0.001	2.50 (1.65-3.78), P <0.001	2.69 (1.80-4.05), P <0.001	2.45 (1.72, 3.49), P <0.001	2.49 (1.75, 3.54), P<0.001	2.24 (1.58, 3.17), P <0.001
AIC (*w*)	26,481 (0)	24,481 (1)	27,462 (0)	12,838 (0)	12,803 (1)	13,577 (0)
Stage group[c]:						
II	3.12 (2.49, 3.91), P <0.001	2.4 (1.84, 3.13), P <0.001	1.72 (1.34, 2.20), P <0.001	3.43 (2.25, 5.24), P <0.001	3.50 (2.31, 5.31), P <0.001	2.00 (1.47, 2.73), P <0.001
III	4.33 (3.54, 5.29), P <0.001	4.06 (3.29, 5.01), P <0.001	2.51 (2.11, 2.98), P <0.001	8.52 (5.72, 12.69), P <0.001	9.04 (6.11, 13.37), P <0.001	4.39 (3.35, 5.74), P <0.001
IV	9.59 (8.01, 11.49), P <0.001	9.51 (7.92, 11.42), P <0.001	6.57 (5.64, 7.65), P <0.001	62.23 (42.27, 91.61), P <0.001	62.7 (42.87, 91.71), P <0.001	29.47 (22.96, 37.84), P <0.001

[a] Reference category for Sex is male.

[b] Reference category for Age group is < 44 years.

[*] Models were stratified by Stage group due to violation of the proportional hazards assumption. Note for colorectal cancer models, Age group also showed some violation of the proportional hazards assumption.

[c] Because Stage group was stratified in the final multivariable 4-year Cox models, HRs for stage variables cannot be provided. Out of interest, we developed models where stage was not stratified and displayed the HRs here. Reference category for Stage group is Stage group I.

Table S11. Summary of univariable 4-year Cox proportional hazards survival models by cancer and staging system

	Lung cancer			*Colorectal cancer*		
	Model 1 (RD-stage)	Model 2 (AJCC-SG)	Model 3 (DoS)	Model 1 (RD-stage)	Model 2 (AJCC-SG)	Model 3 (DoS)
Stage [a]	HR (95% CI), P-value	HR (95% CI), P-value	HR (95% CI), P-value	HR (95% CI), P-value	HR (95% CI), P-value	HR (95% CI), P-value
II	3.12 (2.49, 3.92), P <0.001	2.40 (1.84, 3.13), P <0.001	1.69 (1.32, 2.17), P <0.001	3.60 (2.36, 5.49), P <0.001	3.68 (2.43, 5.58), P <0.001	2.01 (1.48, 2.75), P <0.001
III	4.22 (3.45, 5.16), P <0.001	4.00 (3.24, 4.93), P <0.001	2.45 (2.06, 2.91), P <0.001	8.16 (5.48, 12.15), P <0.001	8.65 (5.85, 12.8), P <0.001	4.05 (3.10, 5.28), P <0.001
IV	9.23 (7.71, 11.05), P <0.001	9.18 (7.65, 11.01), P <0.01	6.23 (5.35, 7.25), P <0.001	54.34 (36.94, 79.93), P <0.001	54.58 (37.35, 79.76), P <0.001	25.87 (20.2, 33.12), P <0.001

[a] Reference category is Stage group I.
